# Nasal Chemosensory-Stimulation Evoked Activity Patterns in the Rat Trigeminal Ganglion Visualized by *In Vivo* Voltage-Sensitive Dye Imaging

**DOI:** 10.1371/journal.pone.0026158

**Published:** 2011-10-19

**Authors:** Markus Rothermel, Benedict Shien Wei Ng, Agnieszka Grabska-Barwińska, Hanns Hatt, Dirk Jancke

**Affiliations:** 1 Lehrstuhl für Zellphysiologie, Ruhr-Universität, Bochum, Germany; 2 Kognitive Neurobiologie, Ruhr-Universität, Bochum, Germany; 3 Bernstein Group for Computational Neuroscience, Ruhr-Universität, Bochum, Germany; The Research Center of Neurobiology-Neurophysiology of Marseille, France

## Abstract

Mammalian nasal chemosensation is predominantly mediated by two independent neuronal pathways, the olfactory and the trigeminal system. Within the early olfactory system, spatiotemporal responses of the olfactory bulb to various odorants have been mapped in great detail. In contrast, far less is known about the representation of volatile chemical stimuli at an early stage in the trigeminal system, the trigeminal ganglion (TG), which contains neurons directly projecting to the nasal cavity. We have established an *in vivo* preparation that allows high-resolution imaging of neuronal population activity from a large region of the rat TG using voltage-sensitive dyes (VSDs). Application of different chemical stimuli to the nasal cavity elicited distinct, stimulus-category specific, spatiotemporal activation patterns that comprised activated as well as suppressed areas. Thus, our results provide the first direct insights into the spatial representation of nasal chemosensory information within the trigeminal ganglion imaged at high temporal resolution.

## Introduction

The trigeminal nerve receives polymodal input from diverse tissues (e.g. cornea, facial skin, vibrissa pad, oral and nasal membranes) and mediates various sub-modalities of somatic sensation: temperature sense, proprioception, discriminative touch, nociception and chemosensation [1]. However, little is known about how the trigeminal ganglion represents these different stimuli, or any of its potential internal processing dynamics (as proposed by [2]), which renders this early stage of the trigeminal system as fascinating as unexplored.

Thus far, only a recent study addressed the existence of a fine-grained somatotopic trigeminal organization using extensive electrophysiological sampling, thereby especially focusing on the localization of vibrissae-responsive cells within the TG [3]. However, the representation of volatile substances in the TG has never been addressed: Vertebrate chemosensation is mainly mediated by the olfactory and the gustatory system as well as the trigeminal nerve [4]. Although the olfactory system is the main detector of volatile substances, the trigeminal system also contributes to the overall gustatory and olfactory sensation since most odorants can be also detected via the trigeminal nerve as demonstrated in animal studies [5]–[9] and human psychophysical examinations [10], [11]. Anosmic patients, only relying on trigeminal function, have lost fine odor discrimination skills and are just able to roughly discriminate between different odor categories [12]. Strikingly, the trigeminal system seems to be extremely selective for certain substances as demonstrated by its ability of even distinguishing molecular stereoisomers [13], [14]. However, since most animal studies recorded from free trigeminal nerve endings, knowledge about any potential underlying spatial mapping could not be derived.

The trigeminal ganglia are located at the base of the skull, ventral to the brain. Possibly for this reason, direct *in vivo* optical access, as a way to simultaneously record space-time trigeminal population activity, has not been attempted so far.

Here we established a decerebration protocol that allows optical recording of population activity from a large region of the rat TG simultaneously. A voltage-sensitive dye was used to visualize TG dynamics at high temporal and spatial resolution, complemented by multi-unit electrophysiological recordings. In this early study, we investigate TG responses to a select set of nasally administered stimuli, including both strong trigeminal agonists (CO_2_, ethanol) and olfactory stimuli. Typical trigeminal agonists evoked localized response patterns that were characteristically modulated over time. In contrast, responses to olfactory stimuli were characterized by their low amplitude and widespread activation, which could hint at possible intraganglionic communication mechanisms.

## Materials and Methods

### Animals

Data were acquired from 35 adult male Wistar rats (Charles River Laboratories WIGA, Germany). All animal experiments were carried out in accordance with the European Union Community Council guidelines, approved by the German Animal Care and Use Committee (application number: AZ 9.93.2.10.32.07.022) in accordance with the Deutsche Tierschutzgesetz (§ 8 Abs. 1 Tierschutzgesetz) and the NIH guidelines.

### Surgical Procedure

**Figure 1:**
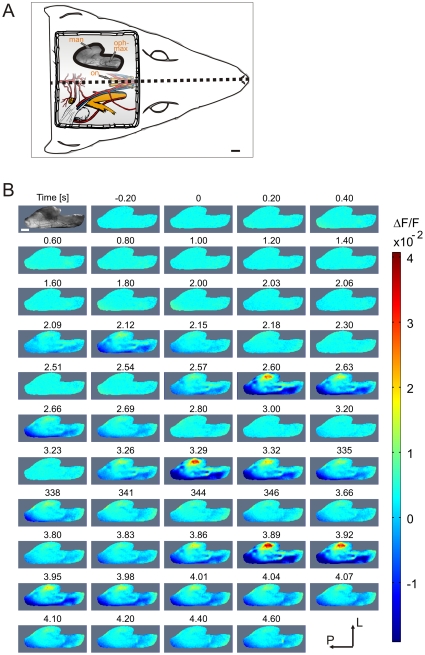
Spatiotemporal dynamics of trigeminal responses in a single trial. (**A**) Schematic view demonstrating TG location at the base of the skull (after decerebration). Bottom: Illustration of the skull-base anatomy (modified from [44]). TG is marked in orange. Top: TG vascular image; scale bar = 1 mm; dotted black line = animal midline; man, mandibular branch; on, optic nerve; oph-max, ophthalmomaxillary branch. (**B**) Timecourse of VSD activity in the TG following nasal application of ethanol. 0 ms denotes stimulus onset. Each frame represents 10 ms of recording extracted from the original timecourse at regular time points (frames of interest are represented in higher temporal resolution). Coordinated activation of the trigeminal ganglion occurred in brief repeated pulses of variable duration and at variable intervals. White bar = 1 mm.

Anesthesia was induced with Chloralhydrate (i.p., 4% solution in saline, 400 mg kg^−1^). Lidocaine (1%, s.c.) was applied to all pressure points and incisions. Immediately before fixation in the stereotactic device, subjects were reverse-tracheotomized (also known as a double tracheotomy, as adapted from an earlier study [6]). In this procedure, two separated tubes were placed into each trachea opening created by a single incision. Subjects were artificially ventilated through the lower tracheotomy tube leading to the lungs (50–70 cycles/min, 4–6 ml tidal volume; UGO BASILE, Italy), while the upper tracheotomy tube allowed for the control of a smooth, constant through-passage of the stimulation air stream. Anesthesia was maintained using isoflurane (1–1,5%). Electrocardiogram and rectal temperature were continuously monitored (core temperature was held at 37.5°C). A craniotomy was performed to expose the cerebral hemispheres, which were then gently aspirated to gain access to the trigeminal ganglia at the base of the skull (**Figure 1A**). After decerebration (which caused unconsciousness) isoflurane was decreased to less than 1% (to rule out influences on nociceptive ion channels). Preparations were stable up to 24 hours. After the experiments rats were euthanized with an overdose of anesthetic.

### *In vivo* VSD loading of the TG

A micropipette was filled with a standard pipette solution containing the VSD (RH-1838, Optical Imaging Inc., NY). A weak manual pressure pulse was used to inject small amounts at multiple ganglionic locations (total injected volume ∼500–1000 nl). The camera was positioned to secure the best possible homogeneous illumination across the ganglionic surface.

### Stimulus application

Stimulation was performed with a custom made olfactometer which contained saturated vapor lines (saturator tubes containing [citral, 1 mM; vanillin, 1 mM; undiluted ethanol; pure CO_2_ (30% final concentration)]) and a control line (saturator tube containing distilled water, 0.4 l/min) that all opened out to a constant clean air background flow (1 l/min) ending in a single tube. Since trigeminal ganglion neurons are polymodal (mechano-, thermo- and chemosensitive) and even their chemosensitivity is not limited to the nasal cavity [15]–[17], we took exceptional care in order to minimize artifacts arising from ectopic and/or mechanical/thermal stimulation: To ensure nasal trigeminal stimulation, the odorant delivery tube was positioned such that it was tightly adjusted to the nostril ipsilateral to the imaged ganglion. To isolate chemosensory stimulation effects, the flow rate (1.4 l/min) and temperature (room temperature) of the stimulation airstream was kept constant throughout the experiment. The use of a constant airflow (as opposed to an artificial sniffing rhythm) further avoids possible mechanical activation of the trigeminal ganglion. Additional steps were taken to minimize artifacts arising from external, mechanical sources such as heartbeat and the respirator (which is decoupled from the stimulation airstream due to the reverse tracheotomy), as outlined in the following subsections.

The stimulus order was pseudo-randomized; inter-stimulus time: 20 sec (between conditions), 3 min (returning to the same condition); pre-stimulus time 200 ms+100 ms olfactometer latency; the stimulus stayed on till the end of the trial.

### VSD imaging and electrophysiology

VSD signals were acquired using a fast CCD camera (Dalstar, Dalsa, Colorado Springs, USA) at 100 Hz (for VSD setup details see [18]) (recording duration = 5 s). Data acquisition was triggered by coincident heart beat and respirator phase, thus minimizing artifacts that could arise from these mechanical events. Their effects are subsequently removed during data processing by filtering and divisive normalization to blank conditions (see below). Post-imaging, we performed targeted electrode penetrations (tungsten electrodes, 1.0 MΩ) guided by the vascular pattern and the imaged activity. The signals were filtered (0.1–0.3 kHz), digitized (25 kHz) and spike sorted to isolate single units (Multiple Spike Detector, Alpha-Omega, Israel).

### Data analysis

In the pre-processing procedure, each acquired pixel was divisively normalized to its mean value during the pre-stimulus period, (i.e. F/F_0_). The activity of each given condition was then compared to the mean of two blank conditions and expressed as relative fluorescence change, where (ΔF/F = F/F_0_−F_B_/F_0B_). Next, the significance of the measured signal across trials was expressed in z-score where z = ΔF/F/√(SEM(F/F_0_)^2^+SEM(F_B_/F_0B_)^2^); SEM = trial-wise standard deviation normalized to the square root of the number of trials. Due to the long recording duration, we sought to eliminate any residual respiration artifact using a frequency filter. In this step, the temporal frequency of respiration-related artifacts was estimated from raw data by applying Fast Fourier Transform to the timecourses of single pixels. Next, a band-stop Butterworth filter (0.5–1.5 Hz) was used to filter out these regular contributions from the pre-processed signal.

Individual activity patterns evoked by each stimulus (“model” patterns) were obtained by initially averaging the main response interval (2 s–4.8 s after stimulus onset) in individual subjects.

In order to determine response onset latencies the model patterns were correlated with z-score values, performed on data from smaller time windows (100 ms). Pearson's correlation coefficients between each 100 ms frame to the corresponding “model” patterns were then studied across animals. The onset latency was defined as a time point at which the correlation coefficients crossed the threshold of 0.8 (the value chosen by comparative analysis including blank condition patterns). Unless otherwise stated, results are presented as mean ± SEM; n = number of animals.

### Virus inoculation and detection procedure

Adapted from [19]. In brief: 5 µl of high titered 5×10^8^ PFU/ml) Pseudorabies virus Bartha strain (PrV-GFP) were inoculated to one nostril of the rat. Animals were sacrificed at different time points after infection in order to visualize viral mediated marker protein expression. An *ex in vivo* preparation of the base of the skull containing the two ganglia [20] was used for marker protein detection. Trigeminal areas displaying infected cells were manually outlined using high resolution epifluorescence images (Zeiss, Axioskop 2, 2,5×, 10×, 20× objective) for each infected animal. We then overlaid outlined areas from different subjects by aligning vascular maps of each ganglion along its midline and the mandibular branch region. The overlay was color coded to indicate the number of animals showing infected areas at identical trigeminal regions.

## Results

### Visualization of evoked trigeminal dynamics using voltage-sensitive dye imaging

We recorded evoked optical signals from the trigeminal ganglion (**Figure 1A**) in response to volatile chemical stimulation. In general, high signal-to-noise ratio was typically attained and in some cases, structured signals could be clearly identified from single trials. **Figure 1B** presents such an example of a single trial optical recording of the trigeminal ganglion (first frame depicts the vascular map) when ethanol was nasally administered (time 0). Coordinated responses in the trigeminal ganglion occurred repeatedly during which a distinct activation pattern was observed. The phasic pattern was jointly expressed by local regions of activation (red areas) as well as suppression of the trigeminal ganglion (blue areas). The ability to visualize individual sweeps in some experiments was an important advantage for characterizing the temporal dynamics of the response to ethanol, because these pulses of activity occur irregularly in time with each stimulus application (see **Figure S1**).

**Figure 2:**
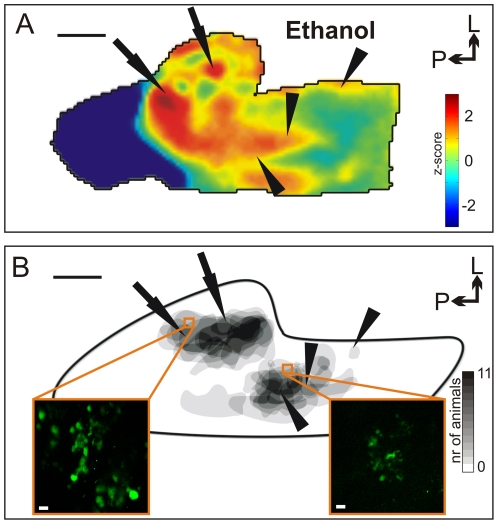
*In vivo* VSD recording of the rat TG. (**A**) Trigeminal VSD activation pattern elicited by nasal ethanol application (z-score map, mean over 2,8 s). Arrow and arrowhead point to activated areas in the near-posterior, central-lateral and anterior trigeminal region, respectively; scale bar, 1 mm; P, posterior; L, lateral (**B**) Correlation of activity patterns to the location of nasal trigeminal neurons: Major clusters of nasal trigeminal neurons could be identified in the near-posterior, central-lateral region as well as in an anterior region on both sides relative to the midline of the ganglion (color code = number of animals showing infected cells at identical trigeminal regions). Arrows point to commonly activated area in CO_2_ and ethanol conditions; arrowheads to unique ethanol activity in VSD measurements (compare **Figure 3A**). Black trace = schematic TG outline; scale bar, 1 mm; Inserts: Representative fluorescence images showing traced nasal trigeminal neurons; scale bar, 20 µm.

For spatial analysis of the evoked activity, we averaged recordings across trials and time. The evoked patterns were then expressed in z-score. Such a statistical activation map derived from a different subject is presented in **Figure 2A**. Activated areas include regions in the near-posterior, central to lateral and anterior ganglion together with suppression in posterior trigeminal areas. Changing the stimulus application site altered this spatial layout: when ethanol was applied instead to the oral cavity, a region in the anterior-medial ganglion became predominantly activated (asterisk **Figure S2**), suggesting minimal (if any) contribution of oral activation to the activation pattern evoked by nasal application of ethanol.

### Evoked activation pattern correlates with afferent neural sources

We then investigated the possible source of these activations using pseudorabies viral tracing since activated regions corresponded in general to the ganglionic localization of trigeminal neurons innervating the nasal cavity – i.e. cells from the nasopalatine/infraorbital nerve, with their somata located in a posterior, central to lateral position in the TG, as well as from the anterior ethmoidal nerve, with their somata located anteromedially [21]. 48 h after unilateral nasal inoculation of the virus, viral-mediated marker protein expression could be detected exclusively in primary infected cells in the ipsilateral ganglion (analog to [20]), demonstrating a specific labeling of nasal TG neurons. Within the ganglion, major clusters of labeled neurons were found in the near-posterior, central-lateral regions (**Figure 2B**, arrows) and in an anterior region on both sides of the ganglion midline (arrowheads, inserts depict traced neurons in the respective regions). Their positions coincided with the evoked activation obtained by VSD imaging, suggesting that they were mediated by cells that receive nasal input.

### Spatial activation depends on the chemosensory stimulus

**Figure 3:**
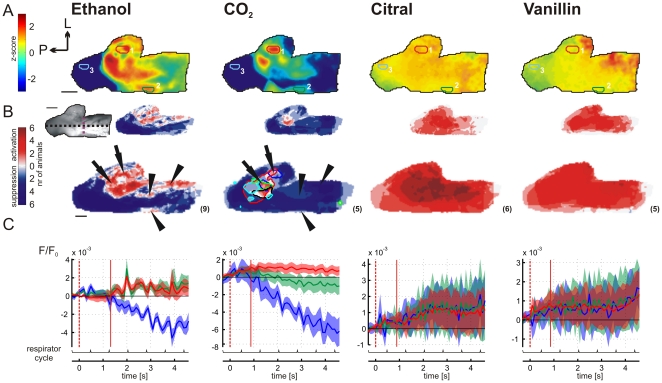
*In vivo* VSD imaging of chemosensory-stimulation evoked activity patterns in the rat TG. (**A**) Representative z-score maps (of a 2000–4800 ms-average) illustrating chemosensory-stimulation evoked TG activity pattern derived from an individual experiment. Citral and vanillin evoked low-amplitude activity across nearly the entire TG; scale bar = 1 mm. P, posterior; L, lateral. (**B**) Activity map comparison across animals demonstrates pattern stability and reproducibility. Overlay color code indicates number of animals showing activation (red) or suppression (blue) at the same trigeminal region (regions that are activated in some animals but suppressed in others offset each other.). The results are presented with two thresholds (z = ±2 (upper row) and ±1 (lower row)) due to a small scatter of activity patterns observed across animals. Contours in the CO_2_ condition (z>1) outline activated regions of individual animals; scale bar, 1 mm. Number in brackets indicates total number of animals for each condition. Arrows point to the common activation in the near-posterior, central-lateral trigeminal area (CO_2_ and ethanol condition). Arrowheads point to the anterior activity, elicited by ethanol but not CO_2_ stimulation (**C**) Local time course (ΔF/F) of activity from the highlighted regions in (**A**) (red trace = local time course from spot 1; green trace = spot 2; blue trace = spot 3, light colored areas = SD across single trials, n = 5 trials). See **Figure S4** for the relationship of each local timecourse to the “blank” (no stimulus) condition. Dotted red line = stimulus onset (300 ms after recording started; stimulus stayed on till the end of the trial). Bottom black trace plots respiration cycle. Vertical red lines indicate response onset latencies.

Next we compared the activity patterns elicited by different stimuli belonging to two categories: substances with a well-known trigeminal component (ethanol and CO_2_) and olfactory stimuli (citral and vanillin). **Figure 3A** shows an example of typical time-averaged statistical activation maps. These were derived from a single animal (same subject as in **Figure 2B**) to which all stimuli were presented (see **Figure 3B** for a summary across different subjects). The location of activated regions by ethanol has been described in the previous section. In the CO_2_ condition, evoked activity was found in the near-posterior, central-lateral regions of the ganglion, together with suppression in broad areas of the posterior and anterior ganglion. The activation pattern was largely similar to the activation pattern evoked by ethanol stimulation, with the exception of lower activation amplitude, particularly in the anterior ganglion. In contrast, olfactory stimuli evoked only moderate levels of activation uniformly across the ganglion with no focal spots of activity or suppressed regions (**Figure 3A**, columns 3 and 4).

To generalize the evoked activity patterns across animals, we overlaid activity maps derived from each subject and condition. These statistical maps were first subjected to a threshold (either z = 2 or z = 1) and then aligned to a common reference point (i.e. the ganglion midline and the mandibular branch region; black horizontal and purple vertical line in **Figure 3B** respectively) derived from the vascular map. Each overlay is color coded according to whether regions were activated (red) or suppressed (blue), and the intensity reflects the number of subjects with commonly activated or suppressed regions. Activity patterns were highly reproducible across subjects, particularly for CO_2_ and ethanol. Arrows point to the common activation in the near-posterior, central-lateral trigeminal area (CO_2_ and ethanol condition) whereas arrowheads point to the anterior activity, elicited by ethanol but not CO_2_ stimulation. In citral and vanillin conditions, all subjects attained at least z-score z = 1, with the majority reaching significance threshold (z = 2; citral: 4 animals of 6; vanillin: 3 animals out of 5). Although undiluted stimuli are often used in human trigeminal psychophysical work [10], and previous studies have pointed to similar trigeminal thresholds in rodents [13] and humans [14], we additionally tested a reduced (50%) ethanol concentration and saturated (20 mM) citral solution for possible concentration effects on the activation patterns. We observed no major effects on the spatial activation pattern (**Figure S3**), although as expected, maximal signal amplitude (ΔF/F) and response latency (see next section) were altered in accordance with the direction of dosage changes (undiluted ethanol 15.6×10^−3^±2.9×10^−3^ n = 5 vs. 50% ethanol 10.2×10^−3^±1.1×10^−3^ n = 4) (20 mM citral 8.8×10^−3^±1.3×10^−3^ n = 3 vs. 1 mM citral 5.1×10^−3^±0.4×10^−3^).

### Differences in the temporal activity structure

**Figure 4:**
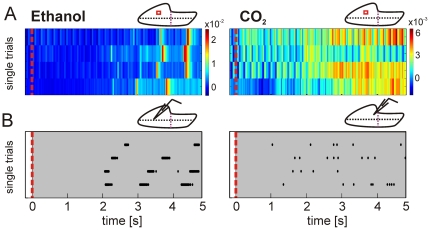
Local VSD activity correlates with suprathreshold electrical activity. (**A**) Single VSD trials obtained from the marked regions in the insert (left ethanol; right CO_2_). (**B**) Raster plots displaying single unit, single trial spiking activity obtained for the marked electrode penetration sites. Note that raster plots (**B**) and single trial VSD activity pattern (**A**) display similar patterns and latencies. Color scale = ΔF/F; red line = stimulus onset.

In order to characterize the evoked dynamics around responsive regions, activity was sampled across regions of interests guided by the layout of the evoked spatial patterns (see colored spots 1/2/3 in **Figure 3A**). **Figure 3C** shows the corresponding local time courses of activity (ΔF/F) at each of these regions (see **Figure S4** for the relationship of each local timecourse to the “blank” (no stimulus) condition). Clear stimulation-dependent modulations were observed only in the ethanol condition, mainly in activated (**Figure 3C**, spot 1 and 2) but also in suppressed regions (spot 3). While ethanol evoked temporally irregular pulses of activation, evoked temporal modulations by CO_2_ as observed in single trials (Fig. 4A), were more regular in average traces (Fig. 3C, second column). Finally, citral and vanillin elicited uniform activation at all three regions without clear temporal modulations (**Figure 3C**, last two columns).

Since activation patterns evoked by different conditions varied greatly in terms of their spatial extent and homogeneity, we applied a pattern-onset latency measurement in order to enable comparison over the entire ganglion for all conditions (for details see Materials and Methods). While the response latency at individual spots can be much shorter (Fig. 3C), pattern-onset latencies were generally long: response onset following stimulation with strong trigeminal agonists were observed after 1500 ms±290 ms; n = 6 (CO_2_) and 2000 ms±210 ms; n = 10 (ethanol) [2900 ms±700 ms; n = 2 (50% ethanol)] and after 1300 ms±290 ms, n = 4 (citral) [800 ms±200 ms, n = 2 (20 mM citral)] and 1800 ms±530 ms; n = 4 (vanillin) for the olfactory stimuli.

### Relation between VSD activity and electrophysiological recordings

To further support the population data reported by VSD imaging, we additionally performed extracellular spike recordings. We inserted electrodes that targeted the near-posterior, central-lateral trigeminal regions (**Figure 4B**, inset) where strong trigeminal agonists evoked the highest activity amplitudes.

It is observed that the temporal structure of spikes and VSD signals are similar. In the case of ethanol, both are characterized by short bursts or pulses of activity (**Figure 4A**, lower left). CO_2_ elicited single spikes that were occasionally clustered over brief time periods (**Figure 4A**, lower right). Further, higher spike counts in each burst corresponded with higher evoked dye amplitudes in ethanol vs. CO_2_ (ΔF/F) (ethanol 15.6×10^−3^±2.9×10^−3^ n = 5; CO_2_ 5.5×10^−3^±0.4×10^−3^; p = 0.01, paired t-test). Even though the population activity reported by VSD imaging and single unit activity recorded by electrophysiology differ with respect to their underlying signal sources, their close correlation is expected from previous studies [18], [22]–[24] and provides support that the obtained VSD signals from the trigeminal ganglion were indeed of neuronal origin.

### Transient suppression of spontaneous activity

**Figure 5:**
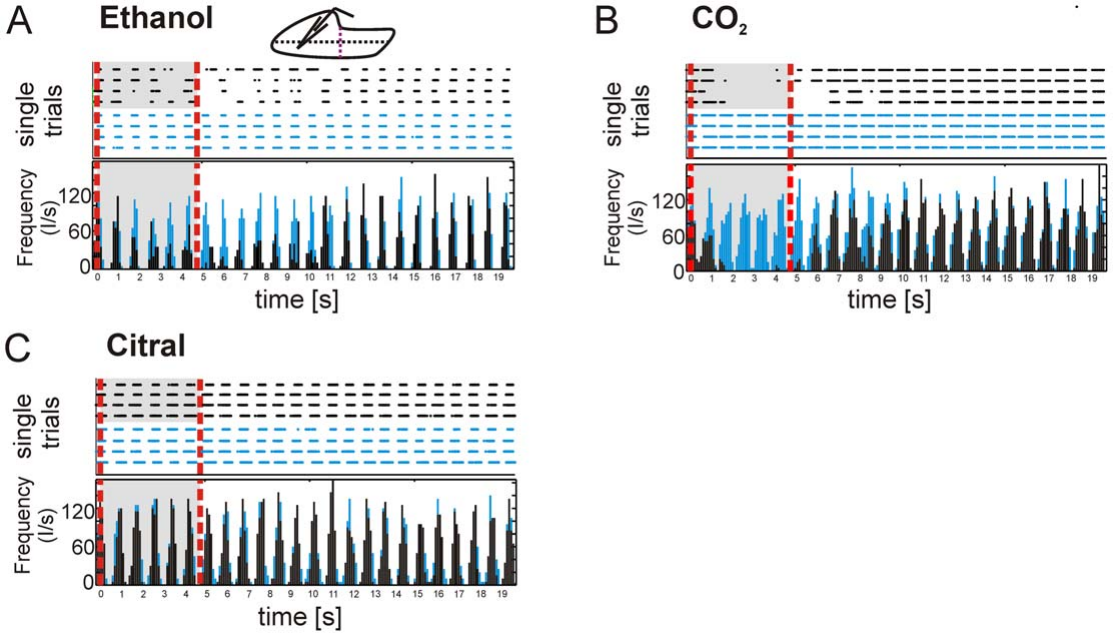
Suppression of ganglionic activity. Raster plots and corresponding Peri-Stimulus-Time-Histogram of spike responses to ethanol (**A**) CO_2_ (**B**) or citral (**C**) application (insert: electrode penetration site) (4 trials; bin factor 100 ms); blue = spontaneous activity; black = stimulus evoked activity; note the rhythmic spontaneous activity found in this region. This spontaneous spiking activity is suppressed by CO_2_ and ethanol application. Recovery from suppression is observed after several seconds. Spontaneous activity is not influenced by citral. Recording duration = 20 s; red lines = stimulus on- and offset.

Interestingly, the strong trigeminal agonists also evoked prominent suppression (as compared to baseline) of the dye signal around near-posterior medial parts of the TG (blue areas **Figure 3A/B**). Electrode penetrations within these areas revealed rhythmic spontaneous spike activity (**Figure 5**, blue) uncorrelated with neither heartbeat nor artificial respiration (switching off the respirator cycles for 30 s did not affect the activity). This spontaneous spiking activity was selectively suppressed when the strong trigeminal agonists were applied (**Figure 5A/B**; black bars) with a mean stimulus induced spike rate reduction of 55.2%±12.9, n = 5. No reduction of spontaneous activity at the same area was observed after citral application (**Figure 5C**).

## Discussion

This study provides the first optical visualization of spatiotemporal activity patterns in the TG. To this end we established an *in vivo* preparation that allows mapping of neuronal activity from the rat TG using high resolution VSD imaging. So far, *in vivo* trigeminal activity studies have been restricted to electrical recordings [3] with limited spatial sampling per subject. Hence, one of the main advantages of our approach is the ability to simultaneously observe neuronal activity across nearly the entire TG; an area about 7 mm long and 4 mm wide.

### Spatial and temporal characteristics of activity patterns

The main trigeminal activity spots observed for the strong trigeminal agonists tested were in accordance with nasal trigeminal projections. This observation was further supported by viral tracing experiments which revealed a large overlap between activated regions observed in the VSD signal and the ganglionic localization of nasal trigeminal neurons.

Why could the tested strong trigeminal agonists then lead to different activation patterns? It is well accepted that subpopulations of trigeminal neurons express different sets of receptor proteins (reviewed in [25]). Trigeminal detection mechanisms for ethanol and CO_2_ are thought to be fundamentally distinct: ethanol affects a long list of voltage- and ligand-gated channels including TRPV1 [26], whereas CO_2_ stimulates free nerve endings through tissue acidification [27], [28]. TRPV1 is activated by extracellular protons [29], [30], and was therefore thought to mediate responses to CO_2_. However, Wang et al., 2010 demonstrated that TRPA1, and not TRPV1 is activated by CO_2_. The differences in activity patterns observed for application of the strong trigeminal agonists are most likely the result of receptor differences within the population of nasal trigeminal neurons. Taken together, while general regions activated by the strong trigeminal agonists seem to be predefined by the somatotopic trigeminal organization, the detailed spatial characteristics of the stimulus-specific activation pattern is likely defined by the individual neuronal receptor/enzyme expression pattern.

Temporally the multiphasic modulations evoked by ethanol are surprising, given that the olfactometer delivers a continuous air flow. External sources of artifacts (e.g. whisker movements, flow-, temperature- fluctuations in the continuous air stream) have been systematically minimized. Additionally, the observed lack of temporal modulations evoked by citral and vanillin and the irregular temporal modulations both within and across single trials for ethanol and CO_2_ (cf. **Figure 4** **and Figure S1**), argue against a mechanical respiration artifact (the start of each imaging trial was coupled to the regular artificial respiration). Instead it is more likely that these multiphasic responses were generated intrinsically by trigeminal neurons. Neurons of the brainstem innervated by trigeminal ganglion neurons have been similarly shown to fire spike bursts spontaneously, as well as at the onset of depolarizing and offset of hyperpolarizing current pulses [31]. It thus seems reasonable to suggest that spatiotemporal activity pattern differences are stimulus-specific and may therefore (as proposed by [31]) serve to encode sensory information in the trigeminal system. In the case of the olfactory system it has already been shown that the precise spatiotemporal arrangement of activity patterns across glomerular structures reflects a fundamental strategy to encode odorant identities [32]–[34].

Another interesting aspect of the observed evoked activity was the relatively long latency of the evoked spatial pattern, e.g. 1500±290 ms (n = 6, CO_2_). However, long latencies were already observed in whole-nerve electrophysiological recordings from the rat ethmoid nerve branch [6]. In addition, although direct comparisons between human psychophysics and animal physiological studies are difficult, an earlier report [35] asking human subjects to track nasal irritation intensity during the continuous presentation of CO_2_ (at a similar concentration used here) also found long delays between stimulus onset and the first non-zero rating (1.63 s; range = 1,34–1,98 s). Additionally, the large variability of onset times in our experiments is in agreement with large individual differences in trigeminal detection times previously reported [35]. Although a direct comparison to our study is difficult, these studies do exemplify a long trigeminal response latency comparable to our findings.

So far applied mainly in the neocortex, VSD signals are known to closely correlate with membrane potential changes while single action potentials are not directly visualized [18], [36]. Nonetheless, there is accumulated evidence that high levels of fluorescent changes signal high probability of spike occurrence [18], [23], [36], [37]. Indeed, regions that were strongly activated in our VSD recordings showed multiunit spiking activity with a similar stimulus-dependent time structure.

In contrast to ethanol (and to a lesser extent, CO_2_), evoked activity by the olfactory stimuli tested here did not feature prominent temporal or spatial modulations. Although in recent years potential molecular receptors for vanillin and citral have been identified, vanillin activates TRPV3 (at high concentrations, 10 mM; [38]) and citral affects TRPV1, TRPV3, TRPM8, and TRPA1 [39], the spectrum of target receptors does not explain the observed homogeneous and broad activation layouts; which also included areas where viral tracing did not reveal any nasal trigeminal neurons (**Figure 2B**). Therefore, our finding for olfactory stimuli is puzzling and certainly needs further exploration in future studies. However, these results were consistently observed across all experiments (see **Figure 3**, 3^rd^ and 4^th^ columns, middle row). Thus, the obtained broad activation patterns might indeed reflect a widespread activation of minor amplitude (local VSD amplitudes (ΔF/F) for citral and vanillin were weaker compared to the other stimuli (citral 5.1×10^−3^±0.4×10^−3^; Vanillin 4.5×10^−3^±0.3×10^−3^ n = 4), involving only few, sparsely-located firing cells. Correspondingly, electrophysiological recordings did not find clear stimulus modulated spikes in response to citral and vanillin: spiking neurons were only rarely detected and if so, spike release was irregular and highly variable across trials. Taken together this may suggest sparse spiking based on close to threshold activation of the TG rather than a simultaneous strong suprathreshold drive of large populations of neurons.

An intriguing speculation is that ectopic trigeminal activation may involve intraganglionic communication mechanisms which would enable chemosensory information processing already at the level of the ganglion. In this regard, TG neurons have been shown recently to directly communicate with satellite glia cells using gap-junctions [2], which are thought to facilitate not just neuron-glia but also glia-glia and neuron-neuron communication. A first attempt to follow-up on these considerations using specific blockers indicated an unlikely involvement of gap-junctions in response to mild trigeminal stimulation (not shown). However, other potential mechanisms of intraganglionic communication, e.g. via GABA, ATP or glutamate or a general involvement of glia cells (reviewed in [40]) can still not be excluded.

### Suppression of ganglionic activity

Investigating spiking activity within suppressed posterior areas in VSD recordings revealed spontaneous rhythmic activity. This ongoing activity was localized given that no such spontaneous firing was found in nearby posterior central regions of the ganglion (**Figure 4B**). The presence of such striking rhythmic activity is certainly intriguing. A possible source of such spontaneous trigeminal activity could be the antidromic transmission [41] of rhythmic spontaneous brainstem activity (pre-Bötzinger region) to cranial nerves, which have been shown to be able to display a rhythm similar to that produced by respiration centers [42], [43]. The specific mechanism that underlies how this spontaneous activity becomes suppressed by stimulation with strong agonists, however, is a detailed topic that is a focus of our future studies.

### Summary

The stimulus-category specific activation patterns described here may indicate the neuronal basis for the well-known trigeminally mediated detection and discrimination abilities of odorous volatiles in humans [12]. In animal models, limited spatial sampling in trigeminal subbranch electrophysiology [6] and interrupted trigeminal connectivity in *in vitro* systems may have so far precluded the characterization of spatially-organized, stimulus-specific and complex responses (involving suppression and broad activation) as demonstrated here using VSD imaging.

Here, we have explored the spatial and temporal characteristics of activity across trigeminal ganglia upon nasal administration of chemical substances *in vivo* and observed clear differences between strong trigeminal agonists and olfactory stimuli. Further investigations of trigeminal representations to an expanded array of chemical stimuli may clarify if suppressive responses are indeed specific for strong (and maybe classified as painful) trigeminal stimuli.

Finally, given that the trigeminal ganglion is not stimulated only by chemosensory input, but indeed receives polymodal input from diverse tissues distributed across large areas of the head (e.g. cornea, facial skin, vibrissa pad, oral and nasal membranes) and can transduce very different sensations, it may be expected that the described preparation should be useful in a broad range of neuroscience-subfields, like barrel field and migraine research.

## Supporting Information

Figure S1 **Series of single trials from a single subject during nasal ethanol stimulation.** As in **Figure 1**, each frame represents 10 ms of activity extracted from the original timecourse at regular intervals, while frames of interest are represented in higher temporal resolution. Each row contains successive frames taken from individual trials. Each frame is color-scaled to the max and min of individual trials to more clearly demonstrate the temporal jitter of evoked activity patterns by ethanol.
(TIF)

Figure S2 **Activation pattern are stimulus site dependent.** Trigeminal VSD activation pattern elicited by nasal (top) or oral (bottom) ethanol application (time-averaged z-score maps, n = 5 trials each). Activity pattern changes with application site: the asterisk points to the broad region dominantly activated by oral compared to nasal ethanol application; color scale = z-score values; green lines = activated areas (z-score >1); gray lines = suppressed areas (z-score <−1); scale bar, 1 mm. P, posterior; L, lateral.
(TIF)

Figure S3 **Trigeminal activation pattern show stimulus specificity.** Trigeminal VSD activation pattern elicited by 50% ethanol (top) or 20 mM citral (bottom) application (time-averaged z-score maps, n = 10 trials 20 mM citral, n = 3 trials 50% ethanol). Activation pattern of the modified stimulus concentrations (reduction for ethanol; increase for citral) are similar to the pattern elicited by used standard concentrations (**Figure 3**). Color scale = z-score values; green lines = activated areas (z-score >1); gray lines = suppressed areas (z-score <−1); scale bar, 1 mm. P, posterior; L, lateral.
(TIF)

Figure S4 **Local time courses of stimulus- and no-exposure (“blank”) conditions.** Local time course (ΔF/F, time in seconds) of activity from the highlighted regions in **Figure 3A**. This plot demonstrates the relationship between individual local time courses plotted in **Figure 3C** and their corresponding blank condition (spots of interest are identical to **Figure 3A**, red trace = spot 1; green trace = spot 2; blue trace = spot 3, light colored areas = SD across single trials, n = 5 trials).
(TIF)
