# Supplementary figures and images for: Nasal Chemosensory-Stimulation Evoked Activity Patterns in the Rat Trigeminal Ganglion Visualized by In Vivo Voltage-Sensitive Dye Imaging
Source: PLoS One. 2011 Oct 19;6(10):e26158. doi: 10.1371/journal.pone.0026158 (PMC3198387; doi:10.1371/journal.pone.0026158)

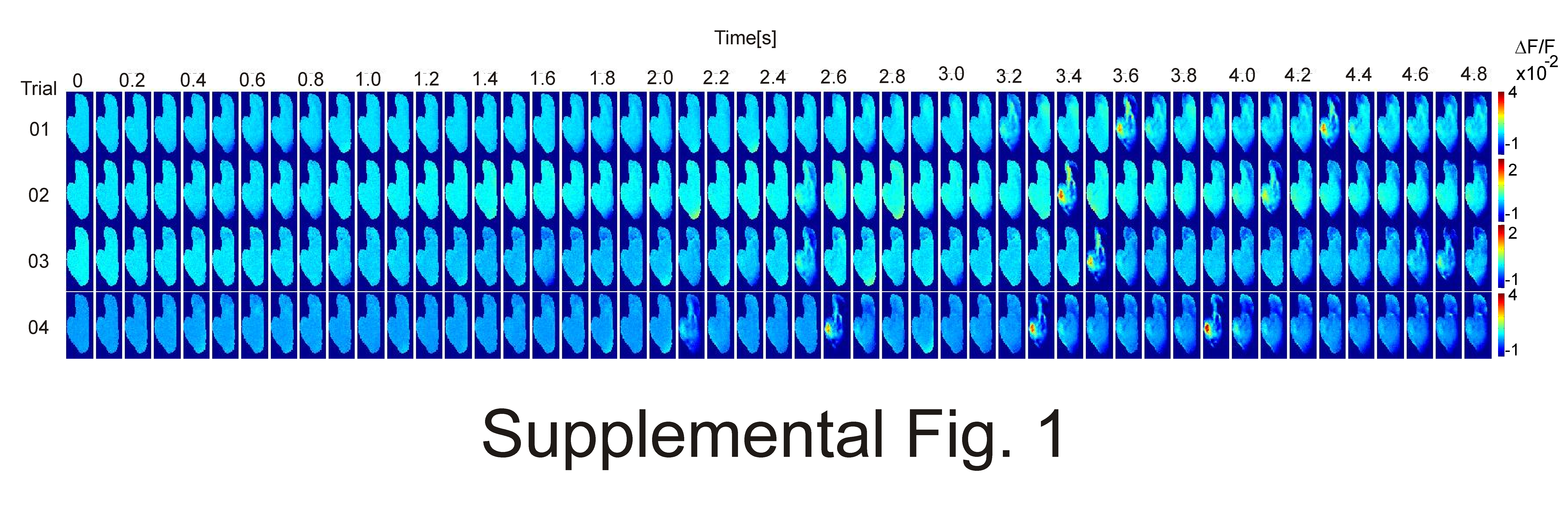

Supplement: Figure S1 — Series of single trials from a single subject during nasal ethanol stimulation. As in Figure 1 , each frame represents 10 ms of activity extracted from the original timecourse at regular intervals, while frames of interest are represented in higher temporal resolution. Each row contains successive frames taken from individual trials. Each frame is color-scaled to the max and min of individual trials to more clearly demonstrate the temporal jitter of evoked activity patterns by ethanol. (TIF) [file pone.0026158.s001.tif]

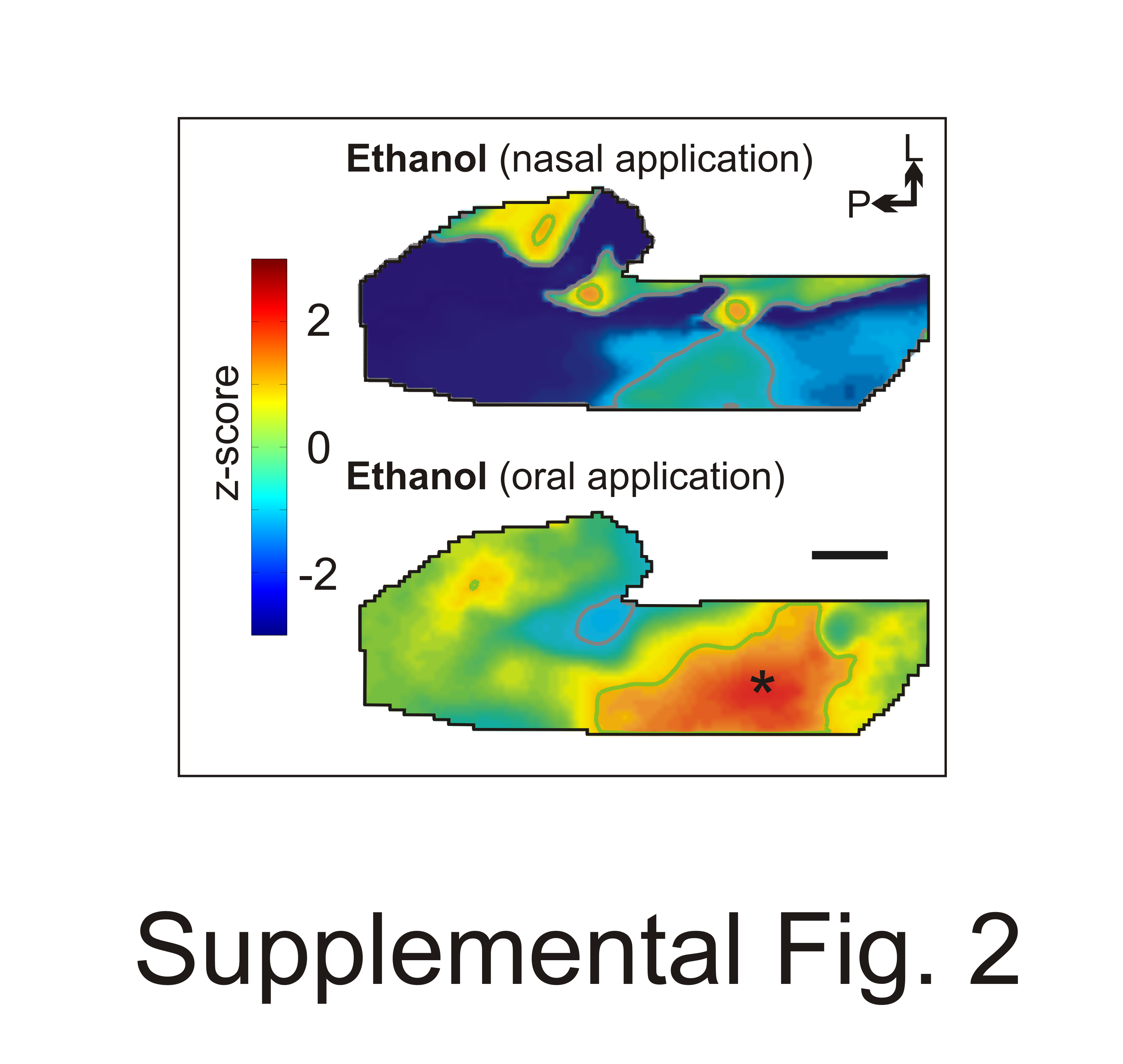

Supplement: Figure S2 — Activation pattern are stimulus site dependent. Trigeminal VSD activation pattern elicited by nasal (top) or oral (bottom) ethanol application (time-averaged z-score maps, n = 5 trials each). Activity pattern changes with application site: the asterisk points to the broad region dominantly activated by oral compared to nasal ethanol application; color scale = z-score values; green lines = activated areas (z-score >1); gray lines = suppressed areas (z-score <−1); scale bar, 1 mm. P, posterior; L, lateral. (TIF) [file pone.0026158.s002.tif]

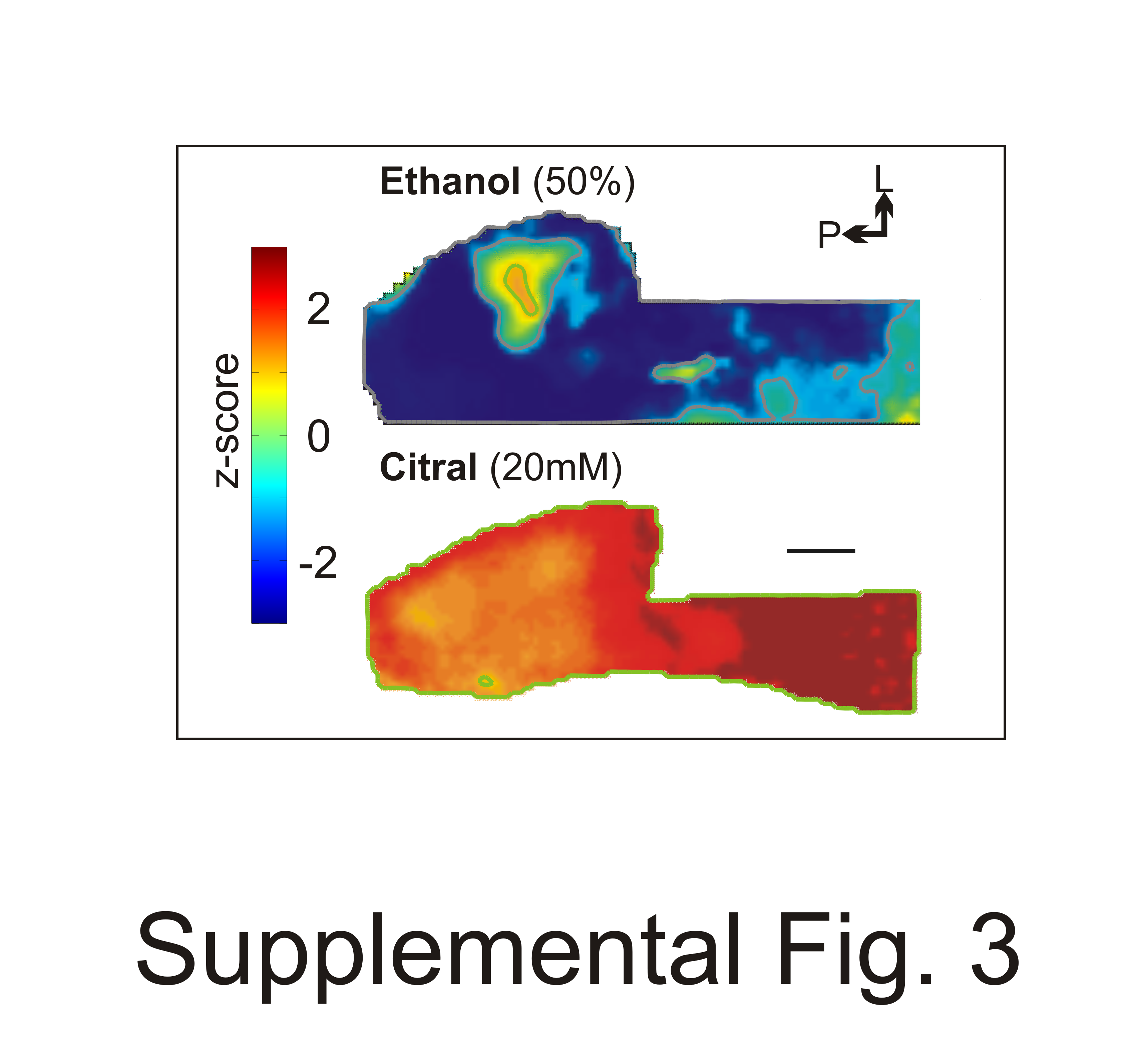

Supplement: Figure S3 — Trigeminal activation pattern show stimulus specificity. Trigeminal VSD activation pattern elicited by 50% ethanol (top) or 20 mM citral (bottom) application (time-averaged z-score maps, n = 10 trials 20 mM citral, n = 3 trials 50% ethanol). Activation pattern of the modified stimulus concentrations (reduction for ethanol; increase for citral) are similar to the pattern elicited by used standard concentrations ( Figure 3 ). Color scale = z-score values; green lines = activated areas (z-score >1); gray lines = suppressed areas (z-score <−1); scale bar, 1 mm. P, posterior; L, lateral. (TIF) [file pone.0026158.s003.tif]

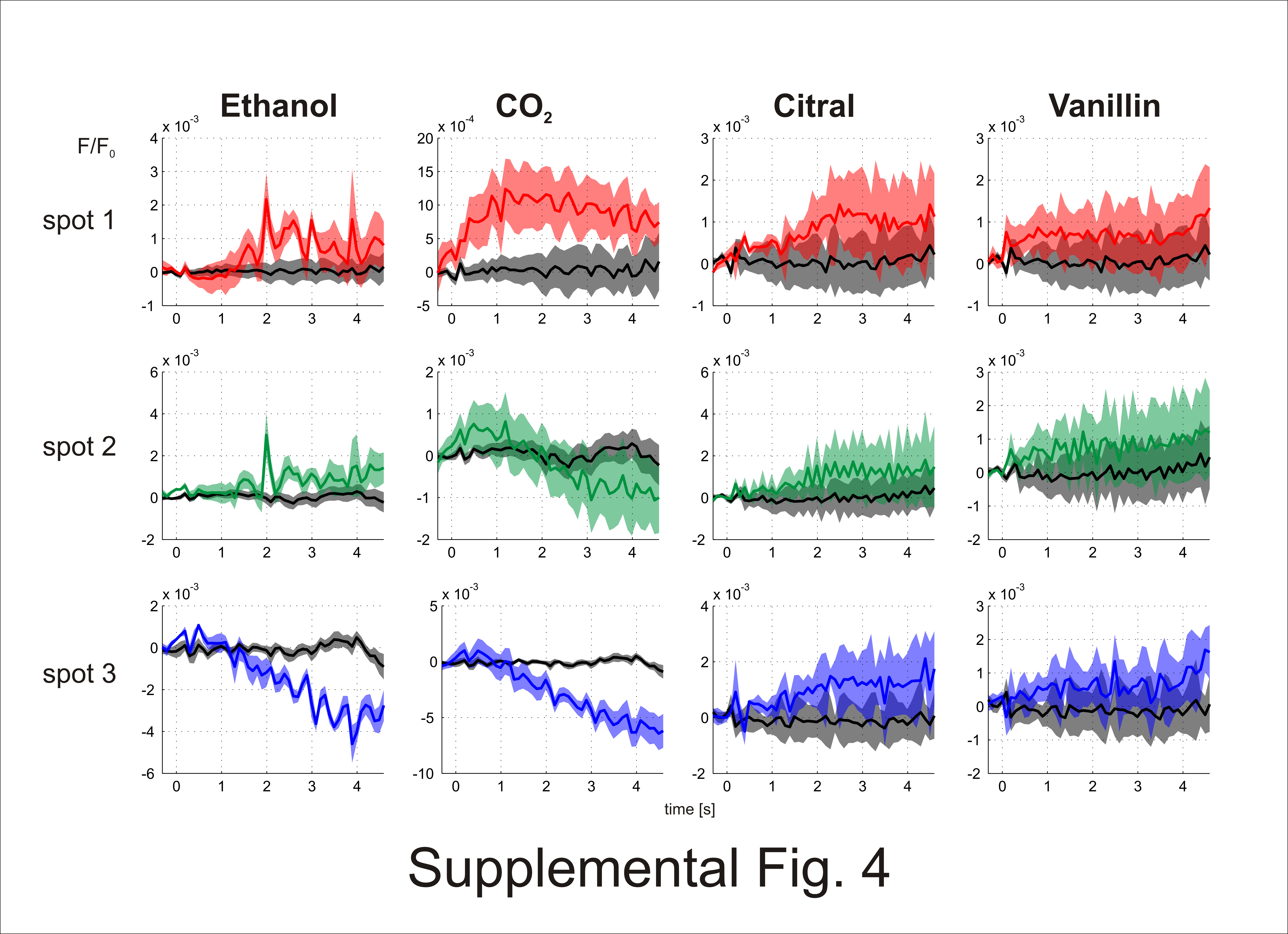

Supplement: Figure S4 — Local time courses of stimulus- and no-exposure (“blank”) conditions. Local time course (ΔF/F, time in seconds) of activity from the highlighted regions in Figure 3A . This plot demonstrates the relationship between individual local time courses plotted in Figure 3C and their corresponding blank condition (spots of interest are identical to Figure 3A , red trace = spot 1; green trace = spot 2; blue trace = spot 3, light colored areas = SD across single trials, n = 5 trials). (TIF) [file pone.0026158.s004.tif]
